# Eye of the future: Unlocking the potential utilization of hydrogels in intraocular lenses

**DOI:** 10.1002/btm2.10664

**Published:** 2024-04-23

**Authors:** Hao Wu, Jiale Wang, Wenhui Fan, Qi Zhong, Rongyue Xue, Siyu Li, Zongming Song, Ye Tao

**Affiliations:** ^1^ Henan Eye Institute, Henan Eye Hospital, People's Hospital of Zhengzhou University Zhengzhou China; ^2^ Zhengzhou University School of Medicine Zhengzhou China

**Keywords:** hydrogel, intraocular lenses, ophthalmic applications

## Abstract

Hydrogels are distinguished by their exceptional ability to absorb and retain large volumes of water within their complex three‐dimensional polymer networks, which is advantageous for the development of intraocular lenses (IOLs). Their innate hydrophilicity offers an optimal substrate for the fabrication of IOLs that simulate the natural lens' accommodation, thereby reducing irritation and facilitating healing after surgery. The swelling and water retention characteristics of hydrogels contribute to their notable biocompatibility and versatile mechanical properties. However, the clinical application of hydrogels faces challenges, including managing potential adverse postimplantation effects. Rigorous research is essential to ascertain the safety and effectiveness of hydrogels. This review systematically examines the prospects and constraints of hydrogels as innovative materials for IOLs. Our comprehensive analysis examines their inherent properties, various classification strategies, cross‐linking processes, and sensitivity to external stimuli. Additionally, we thoroughly evaluate their interactions with ocular tissues, underscoring the potential for hydrogels to be refined into seamless and biologically integrated visual aids. We also discuss the anticipated technological progress and clinical uses of hydrogels in IOL manufacturing. With ongoing technological advancements, the promise of hydrogels is poised to evolve from concept to clinical reality, marking a significant leap forward in ophthalmology characterized by improved patient comfort, enhanced functionality, and reliable safety.

**Translational Impact Statements**

The application of hydrogels in intraocular lenses (IOLs) technology represents a transformative leap forward in ophthalmic medicine. Capitalizing on their superior water retention and biocompatibility, hydrogel‐based IOLs promise to enhance visual outcomes while minimizing postoperative complications. This innovation is poised to offer a new standard for cataract surgery, with the potential to reduce the global burden of vision impairment. As research progresses, hydrogel IOLs may revolutionize patient‐centered eye care, embodying a synergy of advanced material science and clinical expertise.

## 1 INTRODUCTION

**FIGURE 1:**
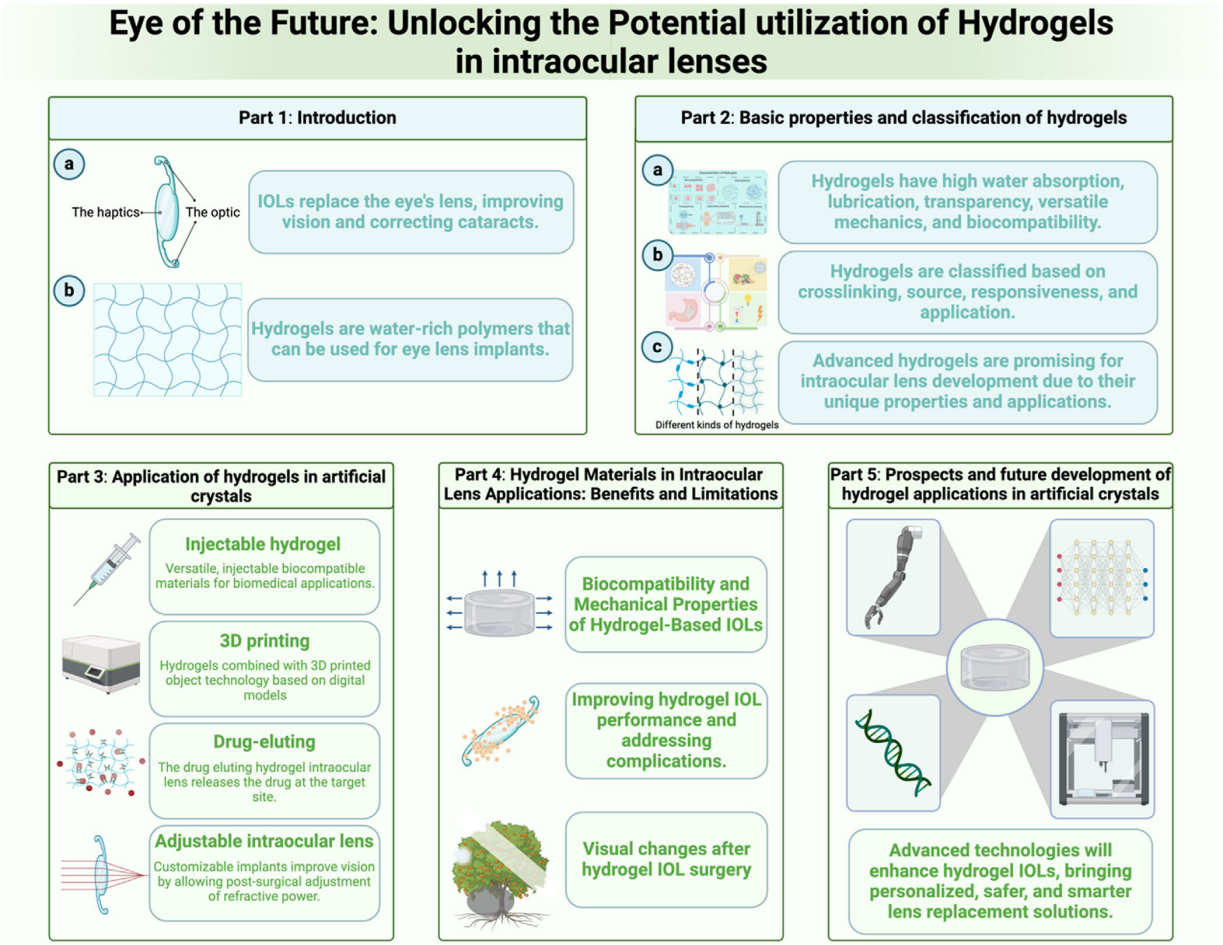
This picture presents a comprehensive schematic illustration delineating the multifaceted potential of hydrogels within the domain of intraocular lenses (IOLs), a pivotal innovation in ophthalmic medical devices. The figure is partitioned into five integral sections, each elucidating a unique aspect of hydrogel application in IOLs, from basic properties to future prospects. The figure starts by explaining how artificial IOLs replace the natural lens to improve vision and treat cataracts, with a focus on the suitability of hydrogels for this purpose due to their unique properties; basic properties and classification of hydrogels: It outlines hydrogels' key features such as high water content and biocompatibility, and classifies them based on their crosslinking, source, responsiveness, and applications, highlighting their potential in the development of IOLs; application of hydrogels in artificial lenses: This part showcases the use of hydrogels in creating lenses for IOLs, including innovative approaches like injectable hydrogels, 3D printing, drug‐eluting capabilities, and adjustable lenses for personalized eye care; hydrogel materials for IOLs: benefits and limitations: It discusses the advantages and challenges of using hydrogel materials in IOLs, emphasizing their biocompatibility, mechanical properties, and the balance between improving IOLs performance and addressing post‐surgery visual changes; prospects and future development of hydrogel applications in IOLs: The final section looks forward to the advancement of hydrogel technologies in IOLs, aiming for more personalized, safer, and smarter lens replacement solutions to meet evolving ophthalmic needs.

Intraocular lenses (IOLs) are pivotal in modern ophthalmology, offering a transformative solution for patients suffering from cataract and refractive vision impairments. These synthetic implants are designed to replace the eye's natural lens when its transparency is compromised by cataract formation or to correct refractive errors, thereby restoring or enhancing vision. The evolution of IOL technology has been marked by significant advancements, with hydrogels emerging as a material of choice due to their unique properties and compatibility with ocular tissues. Hydrogels are polymeric materials characterized by their ability to absorb and retain significant amounts of water within their three‐dimensional networks. These networks are formed by hydrophilic polymer chains, enabling the hydrogels to swell in aqueous environments without dissolving. This unique feature, coupled with their soft tissue‐like consistency, transparency, and biocompatibility, makes hydrogels highly suitable for a wide range of biomedical applications, including drug delivery systems, wound dressings, and, notably, IOLs. The suitability of hydrogels for IOL applications lies in their remarkable similarity to the human eye's natural lens. Their high water content and flexible nature allow them to mimic the physical properties of the natural lens, facilitating seamless integration with ocular tissues. This compatibility significantly reduces the risk of postoperative complications, such as inflammation or fibrosis, enhancing patient comfort and visual outcomes. Moreover, hydrogels' versatility enables the fine‐tuning of their optical and mechanical properties, making them adaptable for various vision correction needs. Hydrogels have been successfully incorporated into several types of IOLs, ranging from soft contact lenses to implantable lenses for cataract surgery. Their application in the field of ophthalmology has revolutionized the approach to treating vision impairments, offering patients a more natural visual experience. For instance, the advent of 3D‐printed hydrogel IOLs allows for a high degree of customization in lens design, enabling personalized eye care solutions that can better address individual patient needs and vision anomalies.^1^ Additionally, the development of injectable hydrogel IOLs introduces a minimally invasive option for lens replacement. These hydrogels can be injected into the eye in a less viscous state and then solidify upon exposure to body temperature or ultraviolet (UV) light, allowing for easier insertion with potentially reduced recovery times and complications.^2^ Despite the significant advancements in hydrogel technology for IOLs, several challenges remain to be addressed. Ensuring the long‐term biostability of hydrogel IOLs, preventing lens opacification, and mimicking the natural lens's accommodation ability are among the primary concerns. Moreover, the interaction of hydrogels with ocular tissues over extended periods requires further investigation to fully understand and mitigate any potential adverse effects. Ongoing research and development are crucial in overcoming these obstacles, with new hydrogel formulations and innovative manufacturing techniques expected to enhance the performance, safety, and efficacy of hydrogel IOLs. In this review, we delve into the potential of hydrogels to revolutionize IOLs, examining their interactions with ocular tissues, assessing current technological advancements, and exploring future applications (Figure 1). With ongoing research and development, new hydrogel formulations are anticipated to emerge, enhancing the efficacy and safety of IOLs. These advancements signal the dawn of a new era in ophthalmology, where the synthesis of innovative materials and surgical techniques promises to improve patient care and treatment outcomes significantly.

## 2 BASIC PROPERTIES AND CLASSIFICATION OF HYDROGELS

### 2.1 Basic characteristics of hydrogels

**FIGURE 2:**
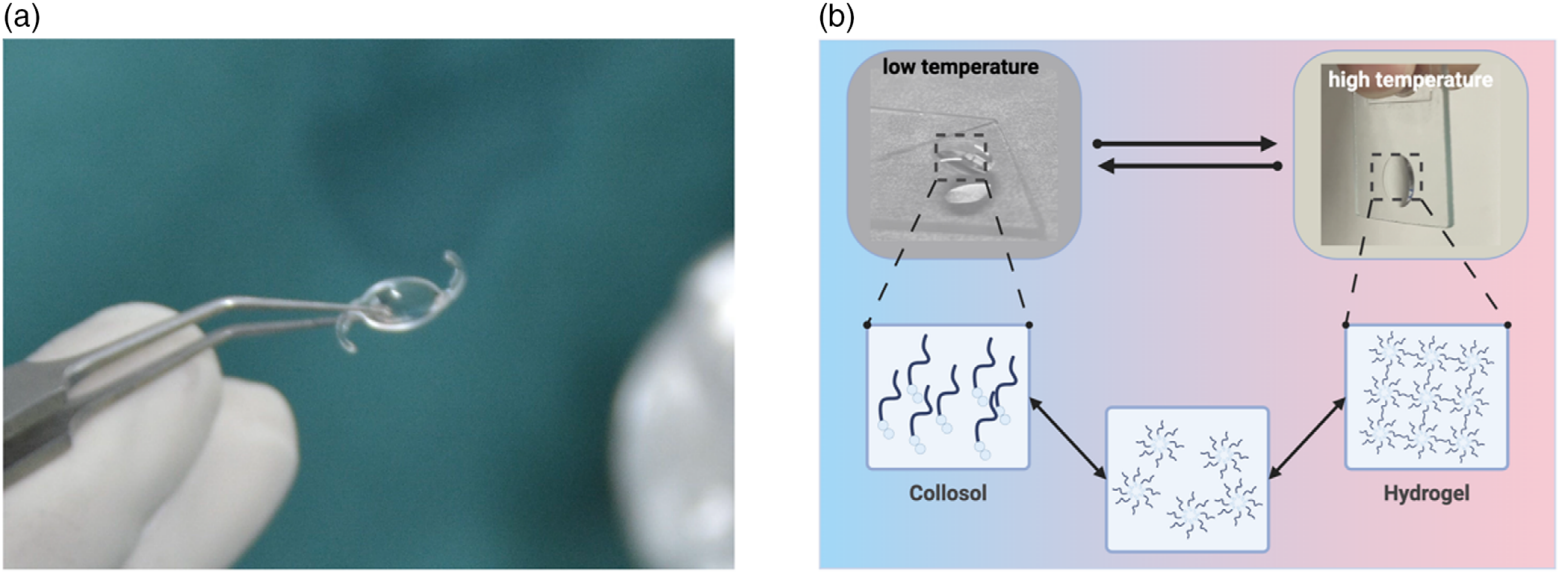
Advanced hydrogel‐based artificial intraocular lens (IOL) and sol–gel transition mechanism. Panel (a) of the figure presents a close‐up view of a state‐of‐the‐art hydrogel‐based artificial IOL delicately held with forceps, demonstrating its physical resilience and optical clarity. This IOL epitomizes the cutting‐edge of biomaterial engineering, designed to mimic the optical properties and mechanical flexibility of a natural human lens. The translucent and pliable nature of the hydrogel material is evident, highlighting its potential for seamless integration within the ocular environment. Such innovations are pivotal in the quest to restore vision to those afflicted with cataracts and other lens‐related impairments, offering a synthetic yet biocompatible solution that promises improved patient outcomes. Panel (b) illustrates the fundamental process of sol–gel transition that underpins the functionality of injectable hydrogel. This thermo responsive behavior is depicted in a schematic that bifurcates into branches to contrast the states at low and high temperatures. On the left, the hydrogel precursor exists as a colloidal solution at lower temperatures, maintaining a low viscosity state that is conducive for injection through minimally invasive procedures. Upon exposure to temperatures similar to those within the human body, the sol transitions into a gel state, as shown on the right. This phase transformation is driven by thermal activation of polymer cross‐linking, resulting in a three‐dimensional network, thereby forming a stable hydrogel.

**FIGURE 3:**
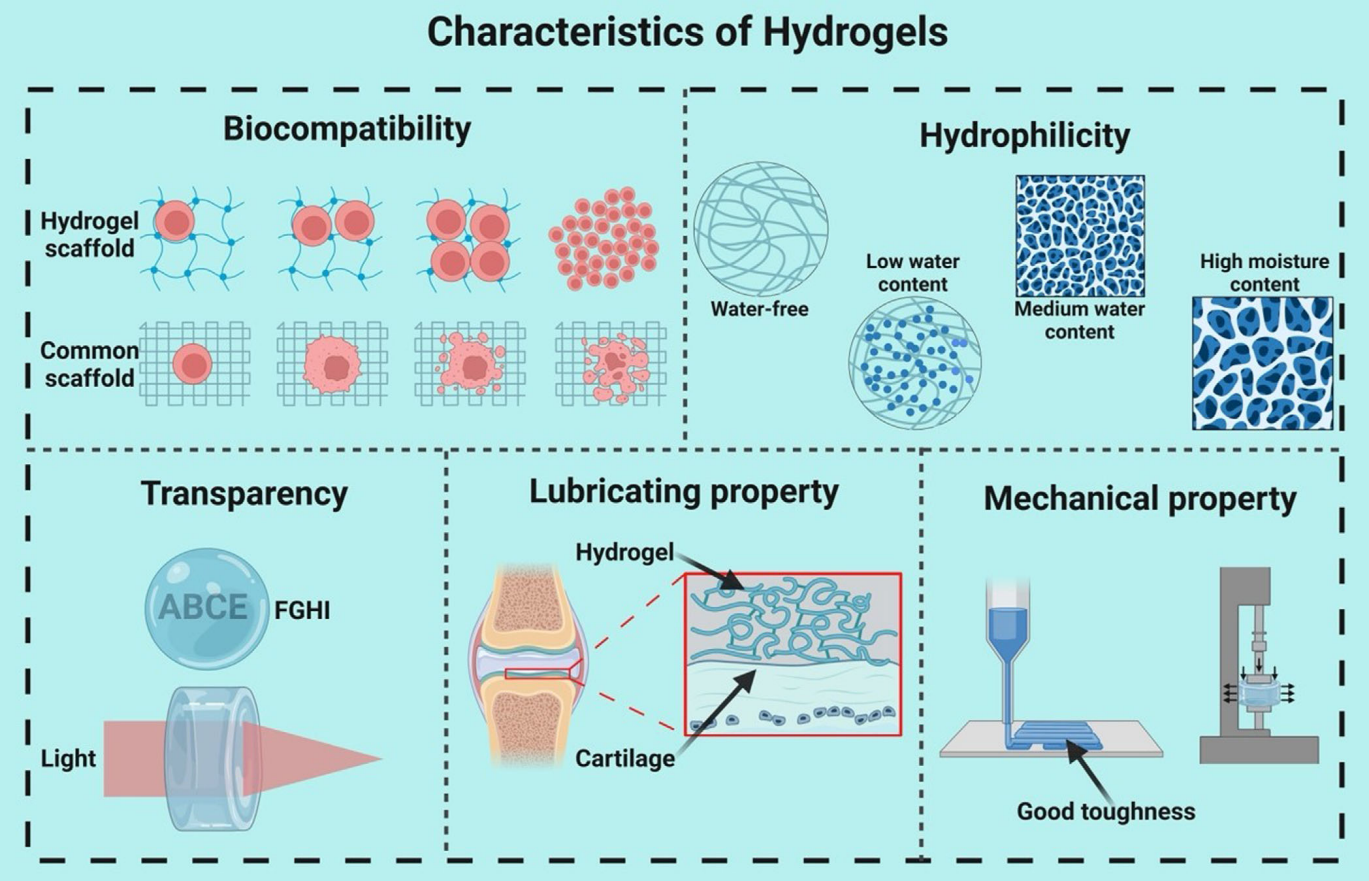
Multifaceted characteristics of hydrogels for ophthalmic applications. This comprehensive diagram encapsulates the quintessential properties of hydrogels that make them suitable for use as artificial lenses in ophthalmic procedures. The figure is segmented into five primary characteristics, each vital to the functionality and compatibility of hydrogels within biological systems.

Renowned for their exceptional characteristics, hydrogel is a category of unique polymers with a three‐dimensional network structure. They can retain large amounts of water and boast a combination of remarkable physical and chemical properties. This makes them an ideal biological material in ophthalmology applications, including producing artificial IOLs (Figure 2a), serving as drug delivery systems, and providing protective coverage for surgical wounds.^3^, ^4^, ^5^ In this context, hydrogel presents a transformative alternative in the biotherapeutics field (Figure 3). First, they exhibit outstanding hydrophilicity owing to their loosely interconnected three‐dimensional polymer network with various hydrophilic functional groups, such as —NH_2_, —COOH, —OH, —CONH_2_, —CONH^−^, and —SO_3_H.^6^ When these groups come into contact with water, they form hydrogen bonds, leading to the swelling of the hydrogel structure. These functional elements enable hydrogels to absorb a substantial amount of water that is even tens of times their own weight. Second, hydrogels are endowed with unique lubricating characteristics. Compared with traditional IOLs materials like polymethyl methacrylate (PMMA), hydrogels offer superior lubrication that can minimize the friction against the delicate tissue of the eye and reduce the likelihood of posterior capsule opacification (PCO).^7^ Due to their biphasic nature, which includes the inability of flexible polymer chains to undergo translational motion and the presence of highly mobile small molecules, the lubrication of hydrogels cannot be described using conventional material‐related terms.^8^ Numerous studies have demonstrated the exceptional lubricating performance of hydrogels, which is of paramount importance in the production of biomedical devices.^9^ Hydrogels can reduce friction‐induced wear and damage, prolong the lifespan of biomedical equipment, and enhance overall efficiency.^10^ However, there exists a trade‐off between lubricity and structural integrity, as too much flexibility can reduce the material's ability to maintain its shape and optical clarity. Furthermore, the transparency of hydrogels is influenced by multiple factors, including polymer molecular weight, concentration, and cross‐linking density.^11^ For ophthalmic applications, hydrogels must possess a high refractive index (RI) above 1.5 to match the refractive power of the natural lens, while maintaining a high water content (>65%) to ensure biocompatibility and hydrophilicity.^12^ Achieving this balance is challenging since hydrogels with higher water content typically have a lower RI, closer to that of water (RI ≈ 1.33). To obtain the necessary RI for producing IOLs, hydrogels should be modified with high RI monomers or by optimizing the water content.^12^ These modifications will not compromise the essential properties of hydrogels. Most hydrogels can be engineered to achieve either high RIs or high water content, making them promising candidates for fabricating advanced biomedical devices.^12^ Hydrogels also demonstrate diverse mechanical properties. The mechanical behavior of hydrogels is binary, exhibiting both viscosity and elasticity.^13^ Hydrogels typically possess excellent elasticity and flexibility. The viscoelastic nature of hydrogels allows them to absorb mechanical stress, which is advantageous for fabricating IOLs since they must withstand constant movement and pressure changes within the eye. Elasticity and flexibility help the IOLs adapt to dynamic ocular conditions without losing its shape. The mechanical strength and stiffness need to be sufficient to maintain the lens structure, while stress relaxation and self‐healing properties can contribute to the durability of the IOLs. The mechanical characteristics of hydrogels, such as mechanical strength, stiffness, stress relaxation, self‐healing, and degradation, can be controlled by introducing specific polymers, comonomers, cross‐linkers, and altering cross‐linking degrees to meet specific requirements.^14^ Finally, hydrogels exhibit perfect biocompatibility, an indispensable feature for any medical material in contacting with biological entities. Perfect biocompatibility means the material does not elicit a significant immune response and can function in contacting with ocular tissue without causing adverse reaction.^15^ Hydrogels used in IOLs are designed to mimic the natural lens environment, thereby minimizing inflammation or rejection. Long‐term in vivo studies are essential to ensure that hydrogels remain biocompatible over the lifespan, without degrading into potentially harmful by‐products.

### 2.2 Classification of hydrogels and their applications

**FIGURE 4:**
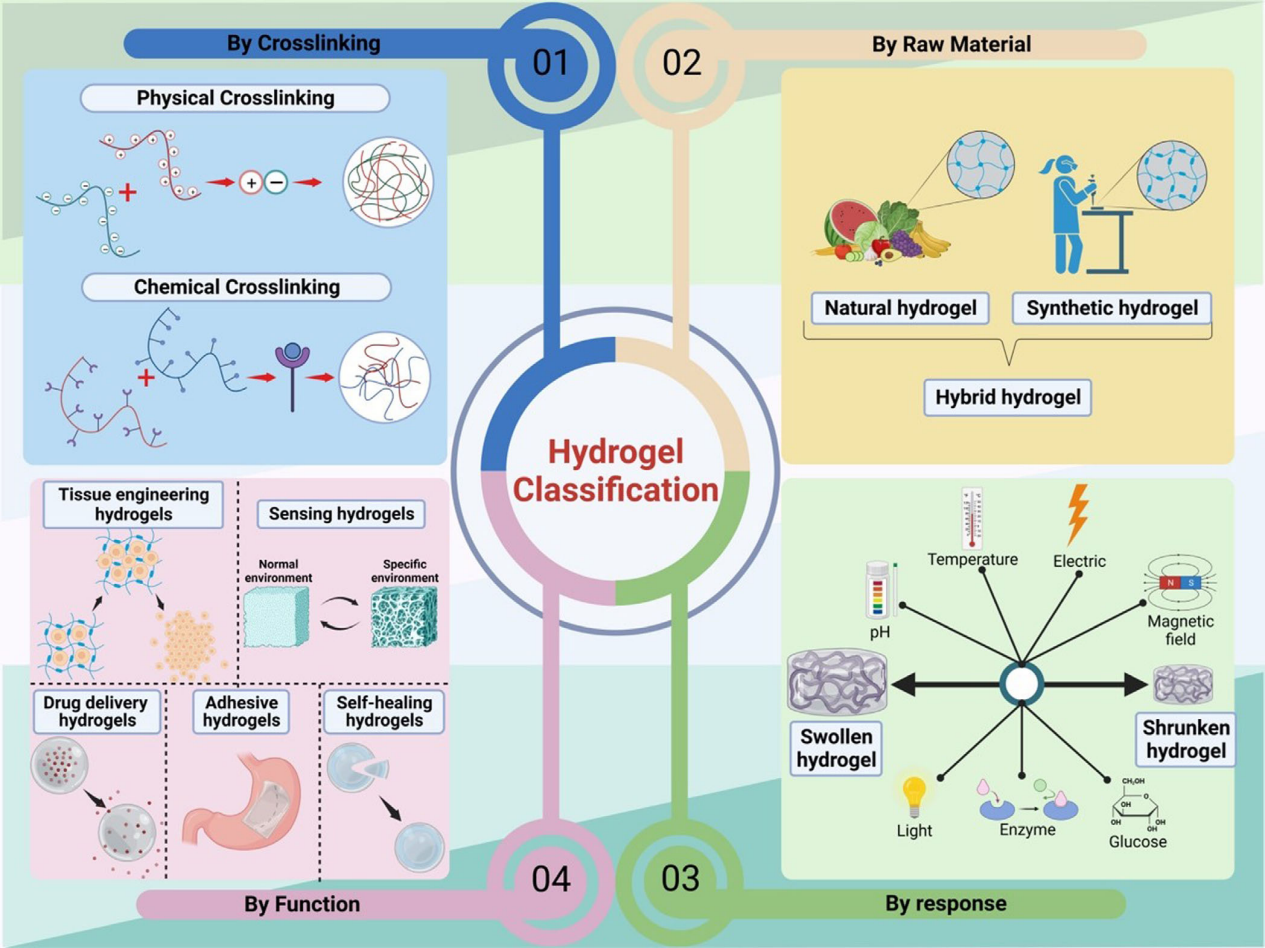
Classification of hydrogels for artificial ocular devices. This schematic provides a comprehensive classification of hydrogels, which are pivotal in the development of artificial ocular devices such as hydrogel‐based artificial lenses. The categorization is multi‐faceted, reflecting the versatility and adaptability of hydrogel materials for ophthalmic applications. Crosslinking mechanisms: The diagram distinguishes between physical and chemical crosslinking mechanisms. Physical crosslinking involves reversible interactions, which are depicted as associations between charged entities, while chemical crosslinking shows covalent bond formation, resulting in a more permanent structure. Origin of materials: Hydrogels are classified according to their origin—either natural, synthetic, or a hybrid of both. This distinction is crucial as it influences biocompatibility, mechanical properties, and degradation behavior, all of which are essential for their function in the eye. Stimuli response: The responsiveness of hydrogels to various stimuli is illustrated, highlighting their smart properties. These materials can swell or shrink in response to changes in pH, temperature, electric fields, magnetic fields, light, enzymes, and glucose levels, showcasing their potential for creating responsive ocular devices that adapt to environmental changes. Functional applications: This section outlines specific applications of hydrogels, such as tissue engineering, sensing, drug delivery, adhesive properties, and self‐healing capabilities. Each application is represented with an icon, suggesting the functional diversity of hydrogels and their ability to address various challenges in ocular tissue engineering and therapeutic delivery.

Thus far, there are several types of hydrogels, each with unique characteristics that make them versatile for different utilizations (Figure 4). They vary markedly in terms of their origins, cross‐linking mechanisms, and responsiveness to external stimuli. First, hydrogels can be divided into physical and chemical categories according to the cross‐linking method.^16^ Physical hydrogels are reversible hydrogels that are formed through intermolecular interactions, such as hydrogen bonds, hydrophobic interactions, or ionic bonds. These hydrogels are held together by molecular entanglements and secondary forces.^17^ The reversibility of these interactions under different environmental conditions endows these hydrogels with unique responsiveness, which can flexible in dynamic physiological environments.^18^ An example of physical hydrogels is agarose gel, widely recognized for its thermal reversibility, where it forms a gel upon cooling and reverts to a liquid upon heating.^19^ Conversely, chemical hydrogels are created by chemical reactions that generate covalent bonds, leading to more stable network structures.^20^ These covalent crosslinks provide a robust and stable network which can withstand external mechanical stress. This stability is critical in applications such as artificial IOLs where long‐term performance is essential. Poly (2‐hydroxyethyl methacrylate) (PHEMA) is a classic example of a chemical hydrogel which has been utilized extensively in ophthalmologic practice for its excellent biocompatibility and clarity.^21^ The choice between these cross‐linking methods depends on the desired hydrogel properties.

Second, hydrogels can be classified into natural hydrogels, synthetic hydrogels, and hybrid hydrogels according to their origins. Natural hydrogels like gelatin, alginate, and hyaluronic acid are primarily derived from biological sources, which is directly obtained from biological systems. These materials possess high biocompatibility and biological activity, making them conducive to biological interactions and cellular integration. For instance, collagen hydrogels have been extensively studied for their ability to facilitate cell migration and proliferation, which are essential for tissue repair processes.^22^ However, natural hydrogels often exhibit relatively lower mechanical strength.^23^ Synthetic hydrogels are composed of synthetic polymers such as polyethylene glycol (PEG), polyvinyl alcohol, polyacrylamide, and PHEMA. These can be custom‐designed to attain specific properties, offering a high degree of control over their physical and chemical characteristics. The versatility of synthetic hydrogels allows for the precise tuning of pore size and degradation rates, making them highly valuable in creating customized drug delivery platforms.^24^ PHEMA, for instance, is utilized in ocular applications due to its transparency and mechanical strength. Hybrid hydrogels are formed by combining natural with synthetic polymers, capitalizing on the advantages of both to create composite materials. These hydrogels aim to synergize the favorable biological interactions of natural polymers with the durability and mechanical strength of synthetic counterparts.^25^

Moreover, smart hydrogels can be categorized into various types based on their responsive behaviors to external stimuli, such as pH‐responsive hydrogels, temperature‐responsive hydrogels, light‐responsive hydrogels, electric and magnetic field‐responsive hydrogels, enzyme‐responsive hydrogels, glucose‐responsive hydrogels, and so on.^16^ These hydrogels exhibit significant changes in volume, shape, and performance under specific external stimulus conditions. For instance, temperature‐responsive hydrogels like poly(*N*‐isopropylacrylamide) undergo a sol–gel transition at their lower critical solution temperature (LCST), making them useful for producing biomedical applications such as controlled drug delivery system and tissue engineering scaffolds (Figure 2b).^26^ Light‐responsive hydrogels, containing moieties like azobenzene, can change their network structure upon exposure to UV or visible light, enabling applications in on‐demand drug delivery systems.^27^ Electric and magnetic field‐responsive hydrogels, embedded with conductive or magnetic particles, can deform or move in response to electromagnetic fields, opening possibilities for soft robotics and targeted drug delivery. Moreover, the enzyme‐responsive hydrogels are designed to react to specific enzymes, leading to structural changes that can be used in drug delivery systems targeting diseases like cancer.^28^ Additionally, the g glucose‐responsive hydrogels can regulate their behavior in response to alterations in glucose levels, offering potential for developing the self‐regulating insulin delivery in diabetes management.^29^ Taken together, each type of these smart hydrogels is engineered to exploit specific interactions or reactions to an external stimulus, thereby fulfilling their complex functions in various medical applications.

Bioelectric signals, generated by the body's biological activities, offer a unique avenue for interfacing natural physiological responses with artificial devices. Among these, electromyogram (EMG) and electrooculogram (EOG) signals are particularly relevant to the advancement of IOL technology. EMG signals, produced by muscle activity, and EOG signals, associated with eye movements, embody the potential to serve as control mechanisms for adjusting the focus of hydrogel IOLs dynamically in response to the visual demands of the user. EMG signals, arising from the muscular activities around the eye, primarily govern the opening and closing of the eyelids and the rotation of the eyeball. Theoretically, these signals could be harnessed to control the focusing mechanism of adjustable IOLs. EOG measures the potential changes associated with eye movements, capturing the electrical field variations as the eye rotates. This capability to detect eye movement directions and amplitudes offers a compelling method to input control signals for IOL functionality. While the concept of utilizing bioelectric signals to augment the functionality of hydrogel IOLs is still in its nascent stages, it holds considerable promise. The integration of such signals could revolutionize the adaptability of IOLs, making them more responsive to the user's visual environment and tasks. However, realizing this potential requires significant advancements in microelectronics, biosensors, and biocompatible materials. The development of hydrogel IOLs that can interface effectively with bioelectric signals is at the forefront of this research, aiming to merge the benefits of hydrogel materials with the innovative application of bioelectricity for a new generation of smart IOLs.

Finally, hydrogels can be further classified based on their specialized applications, encompassing drug delivery hydrogels, tissue engineering hydrogels, sensing hydrogels, adhesive hydrogels, and self‐healing hydrogels. These categories highlight the functional versatility of hydrogels. Drug delivery hydrogels are engineered to release therapeutic agents in a controlled manner.^30^ They can protect drugs from degradation, enhance bioavailability, and provide targeted delivery to specific sites within the body, thereby improving therapeutic efficacy and minimizing side effects. Tissue engineering hydrogels serve as scaffolds that support the growth and differentiation of cells, facilitating the development of new tissue.^31^ They mimic the extracellular matrix, providing a conducive environment for cell attachment and proliferation. Sensing hydrogels are designed to detect and respond to environmental stimuli such as pH, temperature, or the presence of specific analytes.^32^ These hydrogels can be used in diagnostic applications, where they change their physical properties in response to the activities of biological markers, enabling the detection of various health conditions. Adhesive hydrogels are formulated to exhibit strong adhesion properties, making them useful for medical adhesives in wound dressings or surgical sealants.^33^ They can attach to tissue surfaces, provide a protective barrier against infection, and support the natural healing process. Self‐healing hydrogels have the inherent ability to repair themselves after damage, extending their lifespan and functionality.^34^ This property is particularly valuable in dynamic environments where the hydrogel is subject to physical stress, ensuring sustained performance without continuous maintenance or replacement. These classification approaches not only enrich our understanding of the versatile functionalities of hydrogels, but also underscore their potential for innovation in healthcare domains. Each type of hydrogel is tailored to meet the demands of its intended application, demonstrating the material's adaptability and the breadth of its application spectrum.

### 2.3 Advanced hydrogel materials for IOLs

Hydrogels have used to produce the IOLs, on the basis of their unique physical and chemical properties. For instance, PHEMA hydrogels, can be produced by solution polymerization using 2‐hydroxyethyl methacrylate (HEMA) as the primary raw material, ammonium persulfate and sodium metabisulfite as catalysts, along with the cross‐linking additive triethylene glycol dimethacrylate, PHEMA hydrogels exhibit specific biological properties under optimal conditions, including the tensile strength (up to 0.57 MPa), shore A hardness of 23.0, and an equilibrium water content (EWC) exceeding 40%, not to mention over 97% light transmittance.^35^ Remarkably, a recent study shows that manipulating the ratio of hydrophilic monomer HEMA to hydrophobic monomer methyl methacrylate (MMA) would make the hydrogels more suitable for fabricating the IOLs. For instance, a higher proportion of hydrophilic monomers boosts calcium deposits on the hydrogel and increases the EWC (from 16 to 64%). In particular, this manipulation can enhance the transparency (up to 97%) and reduce the hardness (from 92 to 25) of the hydrogel. Even the copolymer's capacity to absorb water decreases after manipulation.^36^ In another insightful study, hydrogels named HAMI—comprising a mix of HEMA, MMA, methacrylic acid (MAA), and HEMA‐indomethacin (IND) were produced using a free radical polymerization approach. These findings demonstrate the versatile advantages of HAMI hydrogels, including the high visible light transmittance, the suitable glass transition temperature (around 70°C) perfect for artificial IOLs biomaterials, the superior mechanical strength (0.57 MPa, Shore A hardness of 23.0), and the excellent biocompatibility with no traces of cytotoxicity. Besides, HAMI hydrogels showcase an impressive EWC exceeding 40%. It can steadily release IND, with a daily dispersal rate that rounds off to approximately 10%.^37^

Recent studies have utilized the poly(acrylamide‐*co*‐sodium acrylate) (PAH) hydrogel for the 3D printing of artificial lenses.^1^ Although the visual quality of PAH as an artificial lens material remains unknown, it has demonstrated satisfactory biocompatibility and strong capacity to inhibit the migrating and proliferating of deleterious cell. These advantages make PAH an ideal transparent biomaterial with maximal optical clarity following implantation.

Poloxamer hydrogel is a biocompatible triblock copolymer composed of poly(ethylene oxide/propylene oxide/ethylene oxide) (PEO/PPO/PEO) units.^38^ This hydrogel consists of 70% hydrophilic PEO units and 30% hydrophobic PPO units, with an average molecular weight of 12,500 Da. Owing to its unique properties and amphiphilic nature, poloxamer hydrogel finds a wide range of applications. When formulated as a 25% Poloxamer hydrogel composition, it exhibits a gelation temperature of 22°C, and has an RI of 1.36, making it a promising candidate for direct lens refilling techniques.^39^ PHEMA/MMA/β‐cyclodextrin (β‐CD) hydrogel is a specially designed and synthesized biomaterial for the controlled release of dexamethasone in artificial crystalline lenses. This type of hydrogels is obtained through thermal polymerization after incorporating the β‐CD. As the β‐CD content increased, PHEMA/MMA/β‐CD hydrogels exhibited a significant rise in the transition temperature from 86.67 to 122.33°C accompanied by the enhanced elastic modulus and tensile strength. During this modulation, the RI increases from 1.463 to 1.466, the elongation at break increases from 40.37 to 60.60%, and the water contact angles decreases from 79.37° to 66.05°. The hydrogels maintained transparency and exhibits high spectral transmittance exceeding 80.0% within the 400.0–800.0 nm visible range.^40^

The research field of wearable epidermal sensors primarily focuses on developing thin, flexible, and breathable materials that can closely adhere to the skin surface for monitoring physiological and biochemical parameters of the human body, such as body temperature, heart rate, blood glucose levels, and so forth.^41^, ^42^ These sensors typically utilize materials including but not limited to conductive polymers, nanomaterials, and silicone gel, which possess excellent biocompatibility, wearability, and sensitivity.^43^ In the realm of IOLs technology, advancements in hydrogel materials derived from wearable epidermal sensor research offer promising innovations for enhancing the functionality and biocompatibility of artificial lenses. The exploration of three distinct hydrogel compositions reveals their potential applicability in developing next‐generation IOLs with improved performance characteristics. First, the self‐adaptive multi‐response thermochromic hydrogel (PHC‐Gel) demonstrates an exceptional blend of mechanical strength, adhesion, and electrical conductivity, thanks to its sophisticated integration of gelatin, PNIPAM, HPC, and CMC.^44^ This hydrogel's ability to achieve ultrahigh transparency and modulate its LCST across a broad range is particularly intriguing. For IOL applications, these features could translate into lenses that adaptively adjust transparency and focus in response to temperature changes, closely mimicking the dynamic responsiveness of a natural lens. Second, the hydrogels (GWHs) designed for Health and Wellness Epidermal Sensors with visible readouts incorporate a poly(acrylic acid)‐poly(acrylamide) network and TA@CNCs, offering repeatable adhesion, thermal compatibility, and long‐term stability.^45^ The incorporation of colorimetric dyes for monitoring physiological and environmental signals suggests the potential for IOLs to not only correct vision but also monitor ocular health indicators, such as changes in intraocular pressure or exposure to harmful UV light, thereby contributing to preventative health measures. Finally, the development of a robust, thermosensitive, and self‐adhesive hydrogel platform (PEST) for thermistor electronics applications highlights a unique balance between flexibility and thermal sensitivity.^46^ The seamless heat conduction interface achieved with this hydrogel could be leveraged in IOLs to enhance the comfort and fit within the ocular environment, ensuring consistent performance without the mechanical mismatching that can occur with traditional rigid lenses. Furthermore, the potential for continuous temperature monitoring introduces an innovative approach to real‐time ocular health surveillance. In conclusion, the integration of these hydrogel materials into IOLs technology could revolutionize the field by introducing lenses that not only improve vision but also adapt to environmental changes, monitor health indicators, and offer enhanced biocompatibility and comfort. These advancements underscore the importance of interdisciplinary research in pushing the boundaries of medical device innovation.

## 3 APPLICATION OF HYDROGELS IN ARTIFICIAL CRYSTALS

**FIGURE 5:**
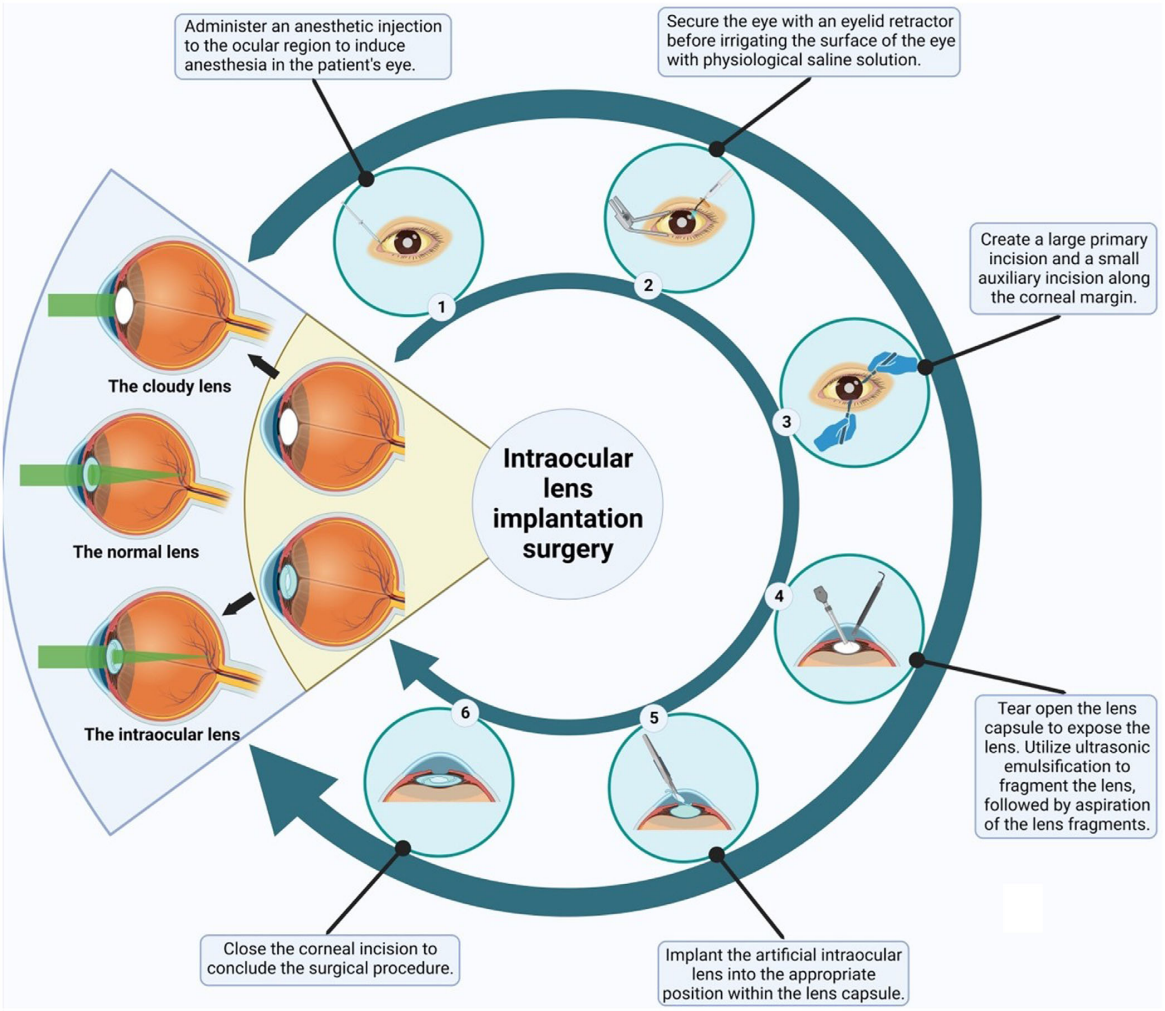
Schematic overview of intraocular lenses (IOLs) implantation surgery. This illustrative figure delineates the sequential steps involved in the surgical replacement of a muddy lens with a hydrogel‐based artificial IOLs, a procedure that restores visual acuity in patients with conditions such as cataracts. The process commences with the administration of an anesthetic to the ocular region, ensuring patient comfort (step 1). The eye is then stabilized using an eyelid retractor and the ocular surface is irrigated with a saline solution to prepare for surgery (step 2). A precise incision is made at the corneal margin to allow access to the lens (step 3). The lens capsule is carefully opened to reveal the compromised lens, which is then fragmented using ultrasonic emulsification and aspirated from the eye (step 4). Subsequently, a pre‐folded hydrogel IOLs is introduced into the lens capsule (step 5). The surgery is concluded by sealing the corneal incision (step 6), initiating the recovery phase where the IOLs integrates within the eye to provide clear vision.

**FIGURE 6:**
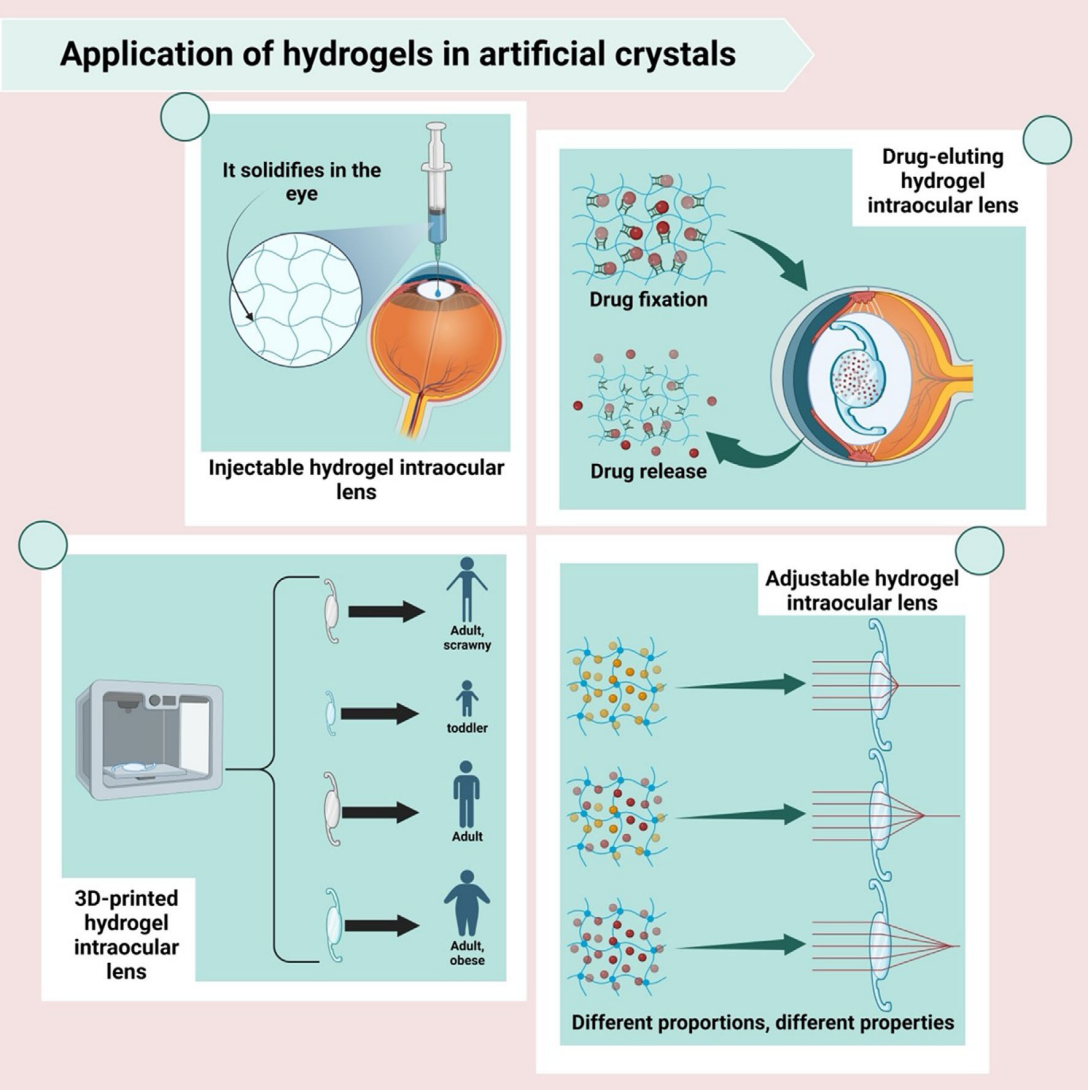
Innovative applications of hydrogels in ophthalmology. This figure illustrates the versatile applications of hydrogels in the development of artificial intraocular lenses (IOLs), showcasing their potential to revolutionize ophthalmic treatments. The top‐left panel depicts an injectable hydrogel IOL, highlighting its ability to solidify upon injection into the eye, adapting to the ocular environment. The top‐right panel details a drug‐eluting hydrogel IOL, demonstrating the dual functionality of such devices in both vision correction and localized drug delivery, with a focus on the mechanisms of drug fixation and controlled release within ocular tissues. The bottom‐left panel introduces a 3D‐printed hydrogel IOL, tailored to individual anatomical requirements, signifying a leap toward personalized medicine in eye care. The bottom‐right panel explores the concept of adjustable hydrogel IOL, where altering the hydrogel's composition can modulate its optical properties, offering customizable vision correction for diverse patient needs.

The crystalline lens of the human eye is a masterwork of biological engineering, comprising a delicate balance of 65% water and 35% protein. With age, the proteins within the lens undergo denaturation, leading inexorably to a diminution in transparency.^47^ This physiological change underpins conditions such as cataracts, a clouding of the lens that obscures vision, and necessitates the surgical intervention to replace the natural lens with IOLs (Figure 5). Since the seminal work of Harold Ridley in 1949, who pioneered the synthesis of the first rigid PMMA IOLs, there has been a relentless pursuit to refine this technology.^48^ Ridley's innovation, while revolutionary, was not without its drawbacks. The rigid nature of PMMA IOLs demanded larger incisions, increasing the risk of postoperative complications such as iris atrophy and secondary glaucoma.^49^ In response to these challenges, the field has witnessed the advent of hydrogel‐based IOLs, materials that have been nothing short of transformative. These flexible and foldable lenses have revolutionized cataract surgery by enabling smaller incisions, thus expediting wound healing and reducing the risk of infection. Moreover, the pliability of hydrogel IOLs has facilitated their insertion into the capsular bag, enhancing the postoperative recovery process and patient comfort. The versatility of IOLs extends beyond their primary function of vision correction; they have been ingeniously engineered to serve as platforms for localized drug delivery. Drug‐eluting IOLs represent a quantum leap forward in ophthalmic postoperative care, offering targeted release of therapeutics to combat infections such as endophthalmitis, thereby attenuating the need for systemic medication and its attendant side effects.^50^ The relentless march of innovation in IOLs technology is underscored by the development of premium lenses that boast an array of patient‐centric features. These include the ability to filter harmful wavelengths, thereby potentially mitigating the risk of macular degeneration, and the incorporation of materials designed to minimize inflammatory responses.^51^ Furthermore, the advent of adjustable IOLs has heralded a new era of personalized ophthalmic care, allowing for postimplantation tweaks to the refractive power to optimize visual outcomes. The escalating demand for tailored solutions underscores ongoing advancements in materials and designs. Due to improvements in biomaterial technology, a wide range of premium IOLs have been developed to filter specific wavelengths, prevent adverse reactions, and allow for adjustment following IOLs implantation. The following section will introduce the latest applications and advancements in hydrogel artificial crystalline lenses (Figure 6) (Table 1).

**TABLE 1:** Advances, challenges, and applications of hydrogels in artificial IOLs.

Hydrogels in artificial crystals	Types of hydrogels	Advancements	Challenges	Solutions and future directions	Applications
3D‐printed hydrogel IOL	PAH hydrogel; GelMA/ChiMA hydrogel	‐ Personalized manufacturing through advanced 3D printing techniques ‐ Improved biocompatibility and transparency	‐ Difficulty in maintaining consistent material properties across different manufacturing batches	‐ Developing standardized protocols for hydrogel synthesis ‐ Investigating the impact of printing parameters on hydrogel properties ‐ Future research could explore multi‐material printing for graded refractive index lenses	Customized IOLs for patients with specific needs
Injectable hydrogel IOL	Poloxamer hydrogel; 4‐PPO/PEO‐based hydrogel	‐ Facilitates minimally invasive surgery ‐ Potential for reducing complications associated with traditional IOL implantation	‐ Mechanisms preventing complications need clearer elucidation	‐ Conducting comparative studies with traditional IOLs to quantify benefits ‐ Exploring the rheological properties of hydrogels to optimize injection	Addressing posterior capsule opacification in cataract surgery
Drug‐eluting and disease detection hydrogel IOL	Poly(HEMA‐*co*‐MMA) hydrogels; HEMA‐IND hydrogels; PHEMA hydrogels; PEGDAAm hydrogels; PEG hydrogels	‐ Dual‐function lenses capable of ocular complication prevention and treatment ‐ Enhanced drug specificity with minimal post‐operative dependency	‐ Complex design leading to potential uncontrolled drug release and compatibility issues	‐ Incorporating feedback mechanisms for controlled drug release ‐ Developing bioresponsive hydrogels for improved compatibility ‐ Future work to focus on patient‐specific drug release profiles	Preventing and treating postoperative complications; monitor certain diseases
IOLs with adjustable hydrogel material	PEG‐PEA/HEMA/styrene hydrogels; 2‐HEMA hydrogels; EOEMA copolymers hydrogels	‐ Adjustable properties to mimic the natural lens ‐ Potential for application in gradient refractive index materials	‐ High manufacturing costs ‐ Technical and clinical translation challenges	‐ Researching cost‐effective manufacturing techniques ‐ Pilot clinical studies to assess efficacy and safety ‐ Long‐term studies on adjustability and stability of hydrogel properties	Enhancing visual quality and adjustability in IOLs

### 3.1 3D‐printed hydrogel IOL

Cataract surgery, the most widely performed ophthalmologic surgery worldwide, involves the removal of a damaged and cloudy lens followed by the replacement with an artificial lens to restore unobstructed vision. Utilizing computer‐aided design and printed biomaterials, 3D printing enables the creation of objects with complex geometries and topologies while preserving their mechanical and biological properties.^52^ Biomedical engineering research has demonstrated the immense potential of 3D printing, particularly in designing the ophthalmology applications such as specialized IOLs for individual cataract patients.^53^ Hydrogels, which are highly absorbent and light permeable due to their high water content, making them ideal materials for fabricating personalized IOLs using 3D printing technology. Researchers have designed a novel responsive PAH hydrogel for precise 3D printing of IOLs molds. Co‐culturing PAH with lens epithelial cells (LECs) or ARPE19 cells did not exhibit any inhibitory effect on cell proliferation. In order to assess its biocompatibility, thin films of PAH were implanted into the eyes of New Zealand white rabbits, while conventional IOLs serving as the control group. The implantation of 3D‐printed IOLs films composed of PAH did not induce any alterations in IOP, blood parameters, ERG responses, or optical structure across all experimental groups. Collectively, these findings suggest that PAH does not induce harm or inflammation in rabbit ocular tissue both in vitro and in vivo, supporting its utilization as a relatively safe material for on‐demand manufacturing of patient‐specific IOLs.^1^ The GelMA/ChiMA hydrogel, when incorporated into 3D‐printed contact lens‐like patches, demonstrates promising potential for application in artificial corneas, offering targeted and sustained release of antimicrobial peptides for effective treatment of bacterial keratitis.^54^ This newly developed 3D printed hydrogel distinguishes itself by its high transparency, excellent biocompatibility, and adjustable mechanical properties, making it an ideal material for personalized and highly comfortable contact lens manufacturing. With these characteristics, the hydrogel has the potential to significantly contribute to the field of IOLs, particularly in enhancing the adaptability and long‐term comfort of such lenses. Nevertheless, IOLs based on 3D printing are confronted with several challenges, such as maintaining appropriate flexibility, high transparency, water richness, UV‐blocking ability and micromechanical characteristics of organic materials. Moreover, researchers should address biosafety concerns associated with IOLs materials.^55^ Therefore, further research is indispensable for improving 3D‐printed hydrogel materials to achieve its wide clinical application.

### 3.2 Injectable hydrogel IOL

The utilization of injectable advanced hydrogel materials for the production of IOLs represents an effective strategy to prevent PCO. Despite advancements in the designment and materials of IOLs, problems related to adaptation loss persist as significant limitations in current cataract surgery. PCO is the most prevalent complication which severely impair the surgical outcomes and visual prognosis of patients. Advances in IOLs and cataract removal technologies have enabled surgeons to perform the minimally invasive surgery via incisions as small as 1.5 mm. However, there is still a need for lenses that can be positioned through even smaller incisions. To address these concerns, injectable IOLs have been developed by pioneering researchers.^56^ By injecting liquid lens material into the capsular bag and allowing it to solidify, a full‐size lens can be created in situ within the eye. This technique offers a marvelous advantage by potentially addressing complications associated with conventional IOLs implantation, such as decentration and PCO. To further enhance the potential of injectable IOLs, a novel nondegradable and fast in situ gelable hydrogel based on 4‐PPO/PEO block copolymer has been developed.^57^ This advanced hydrogel is prepared through a process involving the reaction of 4‐PPO/PEO with epichlorohydrin, followed by a reaction with tyramine, and ultimately formed by an enzymatic cross‐linking reaction. The properties of this hydrogel, such as gelation time and Young's modulus, can be finely tuned by adjusting the HRP/phenol, H_2_O_2_/phenol ratio, and polymer concentration. Remarkably, this hydrogel demonstrates significantly lower LECs attachment compared to commercial IOLs, addressing a key factor in the prevention of PCO. Furthermore, the biocompatibility of the hydrogel is confirmed through tests showing that its gel extract is not harmful to cells, ensuring safety for in vivo applications. The successful use of this hydrogel for in vivo lens refilling in rabbits, with post‐surgery corneal endothelial cell loss and central corneal thickness changes comparable to current IOL implantation procedures, underscores its potential. Histological examinations post‐surgery have shown that the cornea and retina retain their integrity well, indicating that the lens refilling surgery using this hydrogel does not damage these critical eye structures. Notably, Poloxamer hydrogel exhibits remarkable transparency at room temperature and undergoes a sol–gel transition to form a rigid and transparent gel when heated to body temperature. The poloxamer hydrogel, known for its thermal gelation properties and extremely low toxicity, is currently utilized as a carrier for pharmaceuticals and has been studied as an agent in animal models with hyperlipidemia.^58^ Han et al. conducted an evaluation of the compatibility of Poloxamer hydrogel as a manufacturing material for injectable IOLs in vivo and in vitro. In that study, Poloxamer hydrogel suitable for injection presented excellent transparency, and effectively filled the lens capsules with rapidly forming the gel upon reaching body temperature. Even after a period of 3 months following surgery, no signs of PCO were observed. However, it remains unclear whether this absence of PCO resulted from the potential preventative effect of the Poloxamer hydrogel or from the barrier created by its presence or any antiadhesive properties of this material.^39^ Despite the existence of unresolved issues, the utilization of Poloxamer hydrogel has demonstrated promising potential for producing the injectable IOLs as it can be easily delivered into the capsular bag without any risk of leakage post‐injection. Importantly, it can effectively prevent the clouding or opacity formation on the coating surface of IOLs.

### 3.3 Drug‐eluting and disease detection hydrogel IOL

#### 3.3.1 Single‐loaded hydrogel IOL

Postoperative complications including inflammation, infections, and PCO may arise after cataract surgery, posing a threat to the visual recovery of patients. Drug‐eluting IOLs can be employed to inhibit potential infections and other forms of damage to ocular tissues (i.e., avoiding opacification), while simultaneously utilizing their stored drugs for the targeted treatment of some specific lesions as well.^50^ In order to enhance the effectiveness of anti‐inflammatory factors in patients who receipted cataract surgery, Li et al. have synthesized the poly(HEMA‐*co*‐MMA) hydrogels incorporating β‐CD as biomaterials for IOLs, which enabled the loading of dexamethasone. The polymers exhibited high transmittance at visible wavelengths and excellent biocompatibility with mouse connective tissue fibroblasts. Pharmacokinetic studies have shown that the incorporation of β‐CD into hydrogels enhanced the loading efficiency and facilitated the sustained drug release. β‐CD improves the hydrophilicity of the hydrogels as well, leading to a lower water contact angles and greater cellular adhesion to the hydrogel surface. Together, these results suggest that the PHEMA/MMA/β‐CD hydrogels can act as appropriate IOLs materials for in situ drug release of anti‐inflammatory agents post cataract surgery.^40^ Additionally, it has been shown that β‐CD could encapsulate various small molecule therapeutic factors, including antibiotics and antiglaucoma drugs.^59^, ^60^ Thus, IOLs constituted of PHEMA/MMA/ β‐CD hydrogels could be used to treat complicated cases, such as glaucoma with cataract or uveitis cataracts.

Currently, the standard management procedures post cataract surgery involves the utilization of topical antibiotics (typically fluoroquinolones) as a prophylactic measure against bacterial intraocular infection.^61^ To meet these requirements, investigators have designed a novel PHEMA hydrogel that enables sustained and rate‐controlled release of adequate intraocular antibiotics during the immediate postoperative period.^62^ This hydrogel construct is compatible with existing cataract surgical procedures and IOLs implantation techniques. Researchers used the rabbits' model to evaluate the efficiency of IOLs constituted of PHEMA hydrogels. Rabbits in experimental group received the implantation of polymeric device inserted with a standard three‐piece IOLs during surgery, and only received topical steroids after the procedure. On the other hand, the rabbits in control group underwent implantation of conventional IOLs followed by postoperative administration of topical antibiotics and steroids. The results showed that sustained antibiotic concentrations (above the minimum inhibitory concentration for most common bacteria associated with endophthalmitis) were achieved in the eyes of experimental group for more than 4 weeks. The controlled release of antibiotics within the eye provided improved coverage against bacterial infections while reducing patient dependence during postoperative care. These empirical findings provide a new understanding of the antibacterial function of this device.

Early IOLs allowed unrestricted transmission of UV and visible light through the retina, leading to potential damage to the retinal pigment epithelium and subsequent development of retinopathy and age‐related macular degeneration. IND, a nonsteroidal anti‐inflammatory drug (NSAID), has been reported to inhibit mitosis and collagen synthesis in human LECs, thereby preventing PCO.^63^ Additionally, IND possesses absorption spectrum specific for short‐wavelength blue‐violet light. Investigators aimed to design a kind of IOLs loaded with IND as a means to mitigate those postoperative complications and to filter harmful blue light, ultimately improving the treatment prognosis. In this study, I HEMA‐IND were synthesized by esterifying IND and HEMA. Subsequently, hydrogels composed of poly(HEMA‐*co*‐MAA‐*co*‐MMA‐*co*‐HEMA‐IND) were prepared via free‐radical polymerization using HEMA, MMA, MAA, and HEMA‐IND.^37^ Encouragingly, it has been shown that HAMI hydrogels possessed the necessary properties required for IOLs, including filtering harmful short‐wavelength blue light while maintaining visible light transparency, appropriate glass transition temperatures, mechanical strength, and biocompatibility. Furthermore, MAA incorporation enhanced the hydrophilicity of the hydrogels, leading to reduced water contact angle as well as controllable drug release from the hydrogel matrix. Collectively, these findings highlight the great potential of HAMI hydrogels as biomaterials for IOLs to consistently release IND while effectively preventing phototoxicity after cataract surgery.

#### 3.3.2 Dual‐loaded hydrogel IOL

Pseudophakic cystoid macular edema (PCME), resulting from chronic inflammatory response, is the predominant cause of visual impairment in the medium‐term following cataract surgery. The incidence of PCME escalates with the presence of preexisting risk factors, including contralateral PCME, uveitis, diabetes mellitus, retinal vein occlusion, retinopathy, epiretinal membranes, as well as prostaglandin usage.^64^ Adequate pharmacological treatment typically resolves most cases of PCME. Topical steroids have conventionally been used as prophylaxis for PCME in cataract patients. Nevertheless, higher efficacy has been demonstrated for the combined utilization with topical NSAIDs.^65^ Indeed, co‐administration of NSAIDs (e.g., nepafenac or bromfenac) and corticosteroids (e.g., dexamethasone sodium phosphate) has been reported to reduce the incidence of PCME.^66^ The use of topical bromfenac alone or in combination with dexamethasone sodium phosphate has been investigated in an international randomized controlled clinical trial, and again reveals a lower incidence rate with combination therapy.^67^ However, due to the poor penetration of drugs through the corneal epithelium, other methods are required for delivering the less permeative drugs.^68^ In this context, the drug loaded IOLs would provide an efficient approach to deliver drugs without necessitating additional steps during surgery or patient recovery. Nadia Toffoletto et al. employed the incorporation of functional monomers and molecular imprinting to design hydrogels IOLs capable of co‐delivering steroidal (dexamethasone sodium phosphate) and nonsteroidal (bromfenac sodium). In vitro assessment using human LECs has confirmed the non‐toxicity of both the hydrogels and the delivered drugs. The incorporation of *N*‐(2‐aminopropyl) methacrylamide enhanced drug uptake and improved in vitro release kinetics. Imprinting with bromfenac resulted in reduced drug release due to permanent drug bonding, while imprinting with dexamethasone augmented the number of released dexamethasone following dual‐drug loading. A mathematical model was used to predict in vivo drug release behavior, and the results suggested that therapeutic concentrations of bromfenac and dexamethasone could be achieved in the aqueous humor for approximately 2 weeks and several weeks, respectively. These pharmacologic profiles are consistent with current topical prophylaxis after cataract surgery.^4^ Therefore, the dual‐loaded IOLs make up of hydrogels have the potential to effectively replace topical application, address compliance issues, and fulfill an unmet medical need for preventing PCME.

#### 3.3.3 Disease detection hydrogel IOL

Hydrogels, with their high water content and excellent biocompatibility, have emerged as a forefront material in the biomedical field, particularly in applications related to ocular health. These hydrophilic networks, capable of mimicking the natural extracellular matrix, offer a versatile platform for drug delivery and biosensing within the delicate ocular environment. The application of hydrogels in IOLs systems has traditionally been focused on improving post‐surgical outcomes and reducing complications associated with cataract surgery. However, recent advancements have expanded their utility beyond mere vision correction, paving the way for innovative approaches in the early detection and monitoring of intraocular diseases.

One of the groundbreaking developments in this area is the research led by Shin and his team, who have ingeniously modified poly(ethylene glycol) diacrylamide hydrogels by incorporating acrylamide groups to create a fluorescence IOLs.^69^ This novel fluorescence IOL is not just a passive device for vision correction but acts as an active sensor that can detect specific biomarkers associated with ocular health. Their work specifically focuses on the detection of matrix metalloproteinases (MMPs) in the aqueous humor, a group of enzymes known for their role in tissue remodeling and various pathological processes. The ability of this fluorescence IOL to sense MMPs in vivo offers a promising avenue for the early detection of diseases such as Alzheimer's, which is increasingly being linked to ocular biomarkers due to the eye's unique connection to the brain. Building on this concept, further research has led to the development of a PEG hydrogel‐based fluorescent biosensor.^70^ This sensor utilizes photopolymerization techniques to immobilize fluorescein isothiocyanate‐labeled dextran (FITC‐dextran) and tetramethylrhodamine‐labeled concanavalin A (TRITC‐Con A) within the hydrogel network. The design of this biosensor is ingeniously simple yet highly effective in monitoring glucose levels within the ocular environment. In the absence of glucose, the fluorescence from FITC is quenched. However, upon the binding of glucose to TRITC‐Con A, the FITC‐dextran is released, leading to an increase in fluorescence intensity that correlates directly with glucose concentration. This mechanism not only highlights the potential of hydrogels in glucose monitoring but also underscores the broader applicability of hydrogel‐based IOLs in managing and detecting a range of metabolic and degenerative ocular diseases.^71^

The development of such technologies underscores the importance of interdisciplinary collaboration, combining materials science, chemistry, and biomedical engineering to create innovative solutions for complex health challenges. As research in this field continues to evolve, it is expected that hydrogel‐based IOLs will play an increasingly vital role in the early detection, monitoring, and potentially even the treatment of intraocular and systemic diseases, marking a new era in personalized medicine and ocular health.

### 3.4 IOL with adjustable hydrogel material

Currently, extensive research has being conducted to produce IOLs with high‐quality hybrid polymers, such as PEG‐PEA/HEMA/styrene or 2‐HEMA and EOEMA copolymers, with the aim to enhance biocompatibility while simultaneously improving visual quality and adjustability.^72^ These advancements seek to minimize surgical incisions and complications like PCO. For instance, a class of materials known as GRadient INdex (GRIN—with variable RI) has been utilized to optimize the direction of light onto the retina by regulating the variation in RI rather than curvature.^73^ Furthermore, GRIN lens designers can simplify or enhance optical systems using index gradients. By introducing an axial gradient, a spherical surface can replace a traditional lens design requiring an aspheric surface, which significantly simplifies the formation and polishing processes. Even more advantageous are the radial gradient lenses where the RI varies according to the distance from the center of the lens. Radial gradients enable increased focusing power and control over specific aberrations.^74^ Compared with existed techniques employing a single material, the primary advantage of utilizing GRIN‐based IOLs lies in their closer anatomical resemblance to crystalline lenses (which has a rather complex layered structure).^75^, ^76^ Notably, the most significant innovation involves the development of an artificial lens through the combination of multiple materials possessing distinct RIs. Researchers have introduced a new preparation method to address the limitations of conventional hydrogels, such as shortage of combined properties. The IOLs produced by adjustable hydrogel material have a high RI, low modulus, strong flexible strength, and constant transparency.^77^ Raw materials including poly(1‐hydroxy‐1,3‐propylenediyl), poly(ethylene‐*co*‐vinyl alcohol), poly(vinyl alcohol), and poly(allyl alcohol) were cross‐linked with various diisocyanate compounds at low concentrations in a good solvent to form a network. It has been found that the mixture of polymer solutions and cross‐linkers appeared to be critical for achieving transparency. Among them, hydrogels crosslinked with slow‐reacting diisocyanate blocks such as poly(1‐hydroxy‐1,3‐propanediyl) exhibit the most promising properties in terms of transparency, RI, modulus, and tensile strength. The findings would enrich our knowledge of hydrogels and propel its further applications as accommodating lens materials.

## 4 HYDROGEL MATERIALS IN IOL APPLICATIONS: BENEFITS AND LIMITATIONS

**FIGURE 7:**
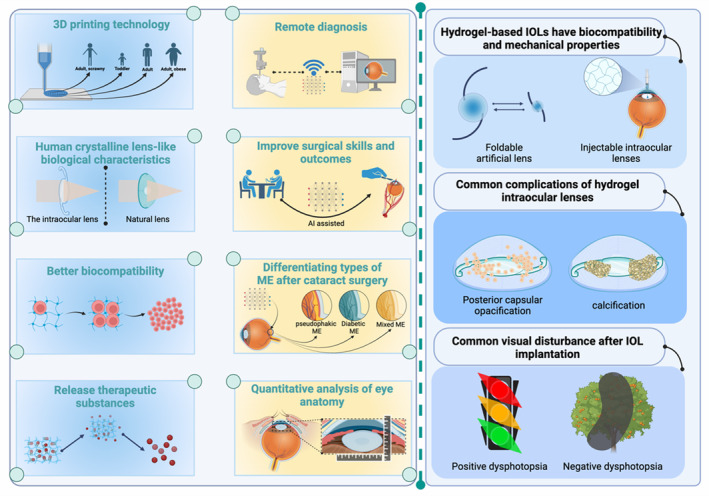
Advancements and considerations in hydrogel‐based artificial intraocular lenses (IOLs). The left section of the figure explores the innovative technologies and biological advantages of hydrogel IOLs. It begins by showcasing the potential of 3D printing technology in customizing IOLs to fit the anatomical diversity across the human lifespan, from toddlers to adults, including individuals with obesity, thus ensuring a personalized approach to lens replacement. Additionally, the figure touches upon the therapeutic potential of hydrogel IOLs, which can be engineered to release medications postimplantation, potentially reducing the need for additional treatments and improving long‐term ocular health. Moreover, the integration of artificial intelligence (AI) in surgical procedures is highlighted as a means to improve skills and outcomes, suggesting a future where AI assists in achieving precision and customization in cataract surgeries. The right section addresses the practical considerations and challenges in the deployment of hydrogel IOLs. It acknowledges the mechanical strengths of hydrogel lenses, such as their foldability and injectability, which facilitate less invasive implantation procedures. However, it also draws attention to common postoperative complications, including posterior capsular opacification and calcification, which remain significant hurdles in the longevity and clarity of IOLs. Finally, the figure addresses common visual disturbances such as dysphotopsia, both positive and negative, that patients may experience after IOL implantation. The depiction of these phenomena underscores the importance of considering patient‐reported outcomes in assessing IOL performance.

The utilization of hydrogel materials in IOLs has brought profound improvements to cataract surgeries, offering distinct advantages and improved visual prognosis. However, these innovative solutions are confronted with potential complications postimplantation. In this context, the following discourse navigates the landscape of hydrogel‐based IOLs, delineating their advantageous characteristics alongside the pressing issues and limitations that arise, particularly focusing on concerns such as posterior capsular opacification, transparency issues, factors influencing the postoperative complications, and dysphotopsias (Figure 7).

### 4.1 Biocompatibility and mechanical properties of hydrogel‐based IOLs

Hydrogel exhibits several advantages in the field of artificial crystalline lenses based on its foldable property. Conventional IOLs, such as hydrophobic acrylic IOLs, have higher rigidity and lower foldability, thereby exacerbating the risk of causing damage to ocular tissue during surgical period. Hydrogels undergo volume reduction in a dehydrated state, rendering them rigid and semi‐transparent. However, upon rehydration (with a water content ranging from 38 to 60%), they can regain flexibility and increase in linear length. This property enables the implantation of dehydrated hydrogel IOLs, which subsequently rehydrate to their original shape at the target location. Consequently, the area of surgical incisions can be effectively reduced, leading to decreased patient trauma and pain levels, shorter healing time, as well as improved safety and efficacy of the surgery. In particular, researchers have devised a novel technique involving the injection of Poloxamer hydrogel as an IOL material into a capsular bag which can be subsequently converted into a solid form. This novel method can form a full‐sized lens in situ within the eyeball, reducing the invasiveness of traditional IOLs implantation and decreasing the occurrence of certain complications, such as dislocation and PCO, thereby offering quicker visual recovery. In specific scenarios, such as post‐YAG laser treatment, hydrogel IOLs exhibit superior suitability compared with traditional PMMA lenses due to their inherent hydrophilic properties, which confer robust resistance against YAG laser‐induced damage. Importantly, the excellent stability and biocompatibility of hydrogel‐based IOLs result in minimal inflammatory response and a non‐adhesive surface within the eyeball. These advantages will promote the smooth development of the hydrogel IOLs and rendering it a safe and reliable choice for cataract patients.

### 4.2 Addressing postimplantation complications and enhancing hydrogel IOL performance

Several potential risks persist after the implantation of artificial crystalline lenses. For instance, hydrophilic hydrogel artificial crystalline lenses are more prone to developing PCO compared with hydrophobic acrylic lenses, which can lead to severe vision impairment. The hydrophilic properties of the hydrogel may result in weak adhesion with the lens capsule, which allows residual epithelial cells to proliferate actively in the retrolental space, whereby clouding the novel implanted lens.^78^ The high prevalence of PCO and the relatively expensive costs associated with Nd:YAG laser capsulotomy make PCO treatment financially burdensome. In this context, the prevention of PCO holds significant medical and economical relevance. Several prophylactic strategies seek to enhance the physical characteristics of IOLs, such as incorporating sharp edges, reducing roughness, minimizing water contact angles, or applying protein/cell‐repellent coatings.^79^ Scaramuzza et al. quantitatively compared the incidence of visually significant PCO and LEC layer formation on the anterior surface of Hydroview hydrogel and AcrySof acrylic foldable IOLs postimplantation. The Hydroview IOLs had round optic edges while Acrysof IOLs had sharp edges. Experimental and clinical studies have shown that the sharp optic edge can prevent the LECs from invading into the retrolental space, thus reducing the incidence of PCO.^80^ Therefore, it is likely that the round optic edges with the Hydroview IOLs could contribute partially to PCO development. Moreover, researchers endeavor to develop novel IOLs materials to prevent PCO altogether. One promising approach involves grafting polymers onto the surface of IOLs to inhibit cell adhesion and proliferation. Bozukova et al. have demonstrated the non‐fouling properties by chemically coating IOLs surfaces with PEG‐chains to prevent cell or protein adsorption.^81^ On the other hand, Lin et al. utilize multilayers of hyaluronic acid/chitosan polyelectrolyte to modify silicone hydrogel IOLs. This adjustment can suppress the primary epithelial cell adhesion and proliferation while maintaining optimal optical properties.^82^

Accumulating evidence suggest that the network structure of hydrogel is water permeable, allowing the intraocular metabolites to penetrate and adhere to it. These metabolites alter the biocompatibility and optic properties of IOLs, leading to reduced transparency and potential impacts on postoperative visual quality. Influencing factors such as the IOLs texture, intraoperative procedures, and comorbidities of patients potentially contribute to or exacerbate this phenomenon. Clinical reports have documented the postoperative turbidity in hydrogel IOLs, with evidence suggesting that calcium deposition plays a significant role in this phenomenon known as “toxic lens syndrome” or calcification. But the precise molecular mechanism underlying IOLs turbidity remains elusive. Consequently, it is imperative to enhance research efforts to elucidate the etiology and mechanisms of IOLs turbidity while concurrently devising preventive strategies against its occurrence.

### 4.3 The potential of hydrogel lenses in reducing dysphotopsias

Dysphotopsias, arise from unwanted patterns overlaying the true retinal image, caused by external light sources.^83^, ^84^, ^85^ These disturbances, often reported as common visual changes post‐IOL implantation, are categorized based on their manifestation and impact on vision into positive dysphotopsia and negative dysphotopsia. Positive dysphotopsia are described by patients as glare, light streaks, starbursts, arcs, halos, or flashes. Negative dysphotopsia as arcuate shadows or lines. Up to 67% of patients develop positive dysphotopsia early after surgery.^86^, ^87^ While these symptoms typically resolve independently, they persist for up to 1 year post‐surgery in up to 2.2% of patients.^88^ The incidence of negative dysphotopsia peaks within the first week post‐surgery, noted by up to 26% of patients^89^; however, symptoms generally persist in 0.13–3.2% of patients 1 year after surgery,^90^, ^91^ and only 1.5% may continue to experience these symptoms 5 years postoperatively.^92^ The occurrence of both positive and negative dysphotopsia is multifactorial, influenced by factors such as the design and shape of the IOL, the sharpness of its edges, and the size of both the pupil and the IOL itself. To date, there is no definitive evidence indicating a correlation between the materials of artificial IOLs and dysphotopsia.^93^ Generally, it is often the structural design of the lens rather than the materials used that can cause dysphotopsia.^93^ However, further evaluation of the role of materials is still warranted. For instance, hydrogel's exceptional mechanical properties and strong processability can contribute to the production of artificial IOLs that do not induce visual impairment. By addressing dysphotopsia and leveraging the material advantages of hydrogel IOLs, surgeons can better meet patients' expectations for restoring high‐quality vision. Future research should continue to explore the long‐term effectiveness of hydrogel IOLs in reducing light disturbances, with a focus on enhancing patient satisfaction and postoperative quality of life.

## 5 PROSPECTS AND FUTURE DEVELOPMENT OF HYDROGEL APPLICATIONS IN ARTIFICIAL CRYSTALS

The integration of artificial intelligence (AI) into ophthalmology, particularly in the domain of cataract surgery and IOLs applications, presents a promising frontier for improving surgical outcomes and patient care. In the preoperative diagnosis and planning process, AI has demonstrated significant potential in remote diagnosis through the utilization of deep learning convolutional neural networks (CNNs).^94^ This technology has the ability to screen and diagnose cataracts using slit lamps and fundus photography. This capability not only facilitates early detection but also aids in the precise preoperative planning necessary for selecting the appropriate hydrogel‐based IOLs. The quantitative analysis of eye anatomy, critical for calculating the power of the IOL, has been traditionally challenging. However, AI algorithms, by analyzing changes in the trabecular‐iris angle and anterior chamber depth post‐phacoemulsification, offer a nuanced understanding that surpasses conventional methods.^94^ This advancement is crucial for hydrogel IOLs selection, where the material's specific properties, such as RI and biomechanical behavior, must be matched with the patient's ocular characteristics to achieve optimal refractive outcomes. The role of AI extends into the operating room, where it transforms cataract surgery through phase recognition and tool detection using CNNs, recurrent neural networks, or support vector machines.^94^ By identifying different stages of the surgery, AI enables the real‐time assessment of surgical skills and decision‐making. This is particularly relevant for hydrogel IOL implantation, where the precision of capsulorhexis and IOL positioning is paramount. AI's ability to provide instantaneous feedback can significantly enhance the surgeon's ability to place hydrogel IOLs accurately, minimizing complications and optimizing visual outcomes. Post‐surgery, AI finds its application in monitoring and managing complications such as PCO, a common issue with IOLs.^95^, ^96^ Machine learning algorithms can differentiate between various types of macular edema (ME) following cataract surgery, a critical aspect in tailoring postoperative care for patients with hydrogel IOLs.^94^ By distinguishing between pseudophakic ME, diabetic ME, and mixed ME, AI facilitates the timely and appropriate intervention, ensuring the long‐term success of hydrogel IOL implantation. The integration of AI into the realm of hydrogel IOLs for cataract surgery is not merely an enhancement of existing procedures but a paradigm shift toward precision medicine in ophthalmology. From preoperative diagnostics to postoperative care, AI's contribution spans the entire surgical continuum, promising improved outcomes, reduced complications, and personalized patient care. As we stand on the cusp of this technological revolution, it is imperative to continue exploring and integrating AI capabilities to fully realize its potential in advancing hydrogel IOL applications in cataract surgery.

In the rapidly evolving landscape of biotechnology and materials science, the research into hydrogels IOLs holds significant promise. The future application of hydrogel IOLs will induce profound advancements and transformative changes. We anticipate several noteworthy developments in the design, production and application of hydrogel IOLs. First and foremost, it is foreseeable that innovative hydrogel materials will progressively exhibit biological properties close to those of natural crystalline lenses. Current research has already shown that hydrogels are capable of mimicking certain characteristics of natural lenses, including adjustable RIs, excellent light transmittance, and flexibility.^35^, ^37^ These features may undergo optimization and enhancement in the future. Second, more efforts should be made to improve the biocompatibility and safety of hydrogel IOLs. On a technical level, enhancing the biocompatibility of hydrogels may lead to shorter periods for lens replacements, significantly improving patients' quality of life. We anticipate that future breakthroughs in material modification could meet more stringent application requirements, such as heightened anti‐inflammation properties and greater stability. Furthermore, with the rapid development of 3D printing technology, our understandings of hydrogels are poised for substantial advancement. Through 3D bioprinting, customized artificial lenses tailored to individual patients' eye size and shape can be created, greatly enhancing the personalized applicability of hydrogel IOLs, and the possibility of seamless, noninvasive implantation of artificial lenses becomes increasingly possible.^1^ Presently, some researchers are exploring for intelligent and dynamically adjustable approaches to produce to innovative hydrogel IOLs. This involves embedding micro‐ and nanoscale photosensitive elements within hydrogels. Such IOLs could dynamically adjust their focus and light transmission in response to changing light intensities, patient age, and near‐to‐far vision requirements. The realization of this concept might introduce a new class of artificial lenses—intelligent, dynamically adjustable IOLs. These innovative hydrogel IOLs could adapt to the ever‐changing conditions of both the human body and the environment, offering an enhanced visual experience. Finally, as colloid science and nanotechnology continue to progress, the fusion of hydrogel research with these disciplines offers a deeper level of integration. Researchers may incorporate active biological molecules such as nanoscale drugs and enzymes into hydrogels, endowing artificial IOLs with potential therapeutic capabilities. In the context of emerging gene therapies and gene editing technologies, we are on the brink of a new era—the advent of gene‐regulating artificial IOLs. This concept could pave the path for a new medical frontier, enabling the targeted treatment of currently incurable ocular diseases. In summary, with their unique physicochemical properties, rich biological functionalities, and exceptional biocompatibility, hydrogels are ideally positioned as the ideal material for producing artificial IOLs. The future application of hydrogels is bright and we are only beginning to scratch the surface of its use in ophthalmologic practice.

## 6 CONCLUSION

Our review has systematically explored the multifaceted nature of hydrogel applications within the realm of IOLs, highlighting their potential to revolutionize the field of ophthalmology. Hydrogels have emerged as a transformative material, boasting properties that closely mimic the natural crystalline lens, including tunable RIs, exceptional light transmittance, and the flexibility required for a more natural visual experience. As we stand on the brink of these groundbreaking developments, it is imperative that we continue to pursue rigorous research and innovation in this field. The integration of hydrogel technology with advanced computational methods, bioprinting, and nanotechnology holds the key to unlocking new frontiers in eye care. It is our hope that this review will inspire continued exploration and development of hydrogel IOLs, contributing to a future where vision impairment is no longer a barrier to a full and active life.

## AUTHOR CONTRIBUTIONS

Conceptualization, H.W. and Y.T.; Writing—original draft, H.W., W.H.F., and J.L.W.; Writing—review and editing, H.W., Z.M.S. and Y.T.; Methodology, H.W., Z.M.S. and Y.T.; Formal analysis, H.W., W.H.F., J.L.W., R.Y.X., S.Y.L. and Q.Z.; Supervision, Y.T. and Z.M.S. All authors have read and agreed to the published version of the manuscript.

## FUNDING INFORMATION

This work was funded by the Health Science and Technology Innovation Outstanding Young Talents Training Program of Henan Province (grant number YXKC2021027), the Basic Research Project of Henan Eye (grant numbers 20JCZD001 and 21JCZD002), the Science and Technology Major Project of Henan Province (grant number 221100310200), and the Central Plains Science and Technology Leading Talent Fund (grant number 224200510013).

## CONFLICT OF INTEREST STATEMENT

The authors declare no conflict of interest.

### PEER REVIEW

The peer review history for this article is available at https://www.webofscience.com/api/gateway/wos/peer-review/10.1002/btm2.10664.

## Data Availability

Data sharing is not applicable.
